# Supplementary material for: Multi‐dataset identification of innovative feature genes and molecular mechanisms in keratoconus
Source: J Cell Mol Med. 2024 Sep 19;28(18):e70079. doi: 10.1111/jcmm.70079 (PMC11412914; doi:10.1111/jcmm.70079)
Supplement: Supplementary file 2 — Material S2. [file JCMM-28-e70079-s002.zip › 7.GSEA analysis/3.DBNDD1/KEGG_STARCH_AND_SUCROSE_METABOLISM.html]

Details for gene set KEGG\_STARCH\_AND\_SUCROSE\_METABOLISM[GSEA]

|  || Dataset | DBNDD1.group.cls#h\_versus\_l |
| Phenotype | group.cls#h\_versus\_l |
| Upregulated in class | h |
| GeneSet | KEGG\_STARCH\_AND\_SUCROSE\_METABOLISM |
| Enrichment Score (ES) | 0.21543051 |
| Normalized Enrichment Score (NES) | 0.64908403 |
| Nominal p-value | 0.9160156 |
| FDR q-value | 1.0 |
| FWER p-Value | 1.0 |
Table: GSEA Results Summary

  

Fig 1: Enrichment plot: KEGG\_STARCH\_AND\_SUCROSE\_METABOLISM      
 Profile of the Running ES Score & Positions of GeneSet Members on the Rank Ordered List

  

| SYMBOL | TITLE | RANK IN GENE LIST | RANK METRIC SCORE | RUNNING ES | CORE ENRICHMENT || 1 | UGT1A8 | na | 313 | 0.502 | 0.0911 | Yes |
| 2 | UGT2B11 | na | 1393 | 0.345 | 0.1288 | Yes |
| 3 | UGT2B4 | na | 2640 | 0.266 | 0.1459 | Yes |
| 4 | UGT1A6 | na | 2660 | 0.265 | 0.1982 | Yes |
| 5 | SI | na | 3810 | 0.220 | 0.2088 | Yes |
| 6 | PYGM | na | 4858 | 0.184 | 0.2154 | Yes |
| 7 | UGT2B28 | na | 6027 | 0.150 | 0.2116 | No |
| 8 | GANC | na | 7271 | 0.118 | 0.1994 | No |
| 9 | GYS2 | na | 8973 | 0.086 | 0.1675 | No |
| 10 | GYS1 | na | 11401 | 0.053 | 0.1082 | No |
| 11 | PYGB | na | 11612 | 0.051 | 0.1123 | No |
| 12 | GAA | na | 12632 | 0.041 | 0.0912 | No |
| 13 | ENPP3 | na | 12822 | 0.040 | 0.0936 | No |
| 14 | PGM2 | na | 13183 | 0.037 | 0.0905 | No |
| 15 | UGT2A3 | na | 13213 | 0.036 | 0.0970 | No |
| 16 | AGL | na | 14060 | 0.030 | 0.0784 | No |
| 17 | UGP2 | na | 17846 | 0.004 | -0.0299 | No |
| 18 | UGT2B10 | na | 17872 | 0.004 | -0.0299 | No |
| 19 | UGT2B15 | na | 18962 | -0.004 | -0.0604 | No |
| 20 | UGDH | na | 19756 | -0.011 | -0.0811 | No |
| 21 | GUSB | na | 20109 | -0.014 | -0.0885 | No |
| 22 | GBE1 | na | 20331 | -0.016 | -0.0917 | No |
| 23 | HK1 | na | 21104 | -0.023 | -0.1094 | No |
| 24 | UXS1 | na | 21733 | -0.029 | -0.1216 | No |
| 25 | PYGL | na | 22898 | -0.042 | -0.1469 | No |
| 26 | PGM1 | na | 23581 | -0.051 | -0.1564 | No |
| 27 | UGT2B7 | na | 24690 | -0.066 | -0.1752 | No |
| 28 | AMY1C | na | 25702 | -0.082 | -0.1880 | No |
| 29 | GPI | na | 25733 | -0.082 | -0.1724 | No |
| 30 | G6PC2 | na | 26032 | -0.088 | -0.1634 | No |
| 31 | HK3 | na | 26158 | -0.091 | -0.1490 | No |
| 32 | ENPP1 | na | 27846 | -0.128 | -0.1722 | No |
| 33 | UGT2A1 | na | 28049 | -0.132 | -0.1517 | No |
| 34 | GCK | na | 28250 | -0.136 | -0.1302 | No |
| 35 | GBA3 | na | 28836 | -0.152 | -0.1167 | No |
| 36 | HK2 | na | 29828 | -0.183 | -0.1089 | No |
| 37 | PGM2L1 | na | 32728 | -0.308 | -0.1311 | No |
| 38 | TREH | na | 32883 | -0.319 | -0.0719 | No |
| 39 | MGAM | na | 34601 | -0.628 | 0.0037 | No |
Table: GSEA details [plain text format]

  

Fig 2: KEGG\_STARCH\_AND\_SUCROSE\_METABOLISM      
 Blue-Pink O' Gram in the Space of the Analyzed GeneSet

  

Fig 3: KEGG\_STARCH\_AND\_SUCROSE\_METABOLISM: Random ES distribution      
 Gene set null distribution of ES for **KEGG\_STARCH\_AND\_SUCROSE\_METABOLISM**
